# 

**Published:** 1880-07

**Authors:** 


### CONTENTS:

PAGE.

ORIGINAL COMMUNICATIONS:

Hints Relative to Intra-Uterine Medication, by James P. White, M. D., . . . . 517
A New Operation for the "Radical" Cure of Hydrocele, by Bernard Bartow, M. D., 527

CLINICAL REPORTS:

A Case of Conservative Surgery. Reported by J. W. Keene, M. D., . . . . . 529
A Death during the Administration of Chloroform, . . . . . . . . 531
Twin Labor—Double Version, by P. W. Van Peyma, M. D., . . . . . . . 532

SELECTIONS:

A New Theory of the Action of Mercury, . 534
A Crucial Test of Homœopathic Medicines, 537
Œdema of the Lungs—Intravenous Injection—Recovery, . . . . . . . 539
Statistics of Cancer in the Breast, . . . . 541
Therapeutic Use of Pilocarpin in Skin Diseases, . . . . . . . . . 543
Action of Various Diuretics, . . . . . 544
Hemorrhoids Treated with Capsicum, . . 544
Cases of Abnormally High Temperature, . 545
Earache and Chloroform Vapor, . . . . 545
Obstinate Hiccough Cured by Muriate of Pilocarpine, . . . . . . . 545
Delicate Test for Albumen, . . . . . 546
Irish Bulls, . . . . . . . 546

EDITORIAL:

Chloroform versus Ether, . . . . . 546
The New Medical Bill, . . . . . . 548
Board of Health Reports, . . . . . 550
Druggists' Association, . . . . . . 552

REVIEWS, . . . . . . . 553

